# INHBA is a mediator of aggressive tumor behavior in HER2+ basal breast cancer

**DOI:** 10.1186/s13058-022-01512-4

**Published:** 2022-03-05

**Authors:** Moqing Liu, Rebecca Smith, Tiera Liby, Kami Chiotti, Claudia S. López, James E. Korkola

**Affiliations:** 1 Department of Biomedical Engineering, Oregon Health & Science University, Portland, OR USA; 2 Department of Molecular and Medical Genetics, Oregon Health & Science University, Portland, OR USA; 3 Multiscale Microscopy Core, Oregon Health & Science University, Portland, OR USA

**Keywords:** HER2+ breast cancer, Lapatinib, Resistance, Basal subtype, INHBA

## Abstract

**Background:**

Resistance to HER2-targeted therapeutics remains a significant clinical problem in HER2+ breast cancer patients with advanced disease. This may be particularly true for HER2+ patients with basal subtype disease, as recent evidence suggests they receive limited benefit from standard of care HER2-targeted therapies. Identification of drivers of resistance and aggressive disease that can be targeted clinically has the potential to impact patient outcomes.

**Methods:**

We performed siRNA knockdown screens of genes differentially expressed between lapatinib-responsive and -resistant HER2+ breast cancer cells, which corresponded largely to luminal versus basal subtypes. We then validated hits in 2-d and 3-d cell culture systems.

**Results:**

Knockdown of one of the genes, INHBA, significantly slowed growth and increased sensitivity to lapatinib in multiple basal HER2+ cell lines in both 2-d and 3-d cultures, but had no effect in luminal HER2+ cells. Loss of INHBA altered metabolism, eliciting a shift from glycolytic to oxidative phosphorylative metabolism, which was also associated with a decrease in tumor invasiveness. Analysis of breast cancer datasets showed that patients with HER2+ breast cancer and high levels of INHBA expression had worse outcomes than patients with low levels of INHBA expression.

**Conclusions:**

Our data suggest that INHBA is associated with aggressiveness of the basal subtype of HER2+ tumors, resulting in poor response to HER2-targeted therapy and an invasive phenotype. We hypothesize that targeting this pathway could be an effective therapeutic strategy to reduce invasiveness of tumor cells and to improve therapeutic response.

**Supplementary Information:**

The online version contains supplementary material available at 10.1186/s13058-022-01512-4.

## Background

ERBB2/HER2 (HER2) amplification occurs in approximately 20% of breast cancers, leading to overexpression of the HER2 protein on the cell surface and aberrant pro-tumorigenic signaling through MAPK and PI3K pathways [1–3]. Multiple studies have shown that HER2+ overexpression in breast tumors is associated with poor outcome and aggressive disease [1–3]. Expression profiling-based classifications confirm that HER2+ tumors represent an aggressive breast cancer subset, in which patients have worse outcomes than patients with other molecular subtypes, such as luminal breast cancers [4, 5].

The high levels of overexpression of HER2 combined with its location on the cell surface led to recognition of HER2 as an attractive target for therapeutic intervention. Efforts to develop anti-HER2 therapeutics resulted in the development of multiple anti-HER2 agents including antibody-based (Trastuzumab, T-DM1, pertuzumab) and small molecule (lapatinib, neratinib, tucatinib) inhibitors that have been approved for use in patients [3, 6–9]. While these have significantly improved patient outcomes, there still exists significant de novo and acquired resistance against HER2-targeted therapeutics that limit their efficacy [10, 11]. Indeed, patients with metastatic HER2+ disease who receive the current approved therapies have an overall life expectancy of ~ 4.5 years [11]. Numerous mechanisms have been suggested to explain resistance, including activation of alternative receptors, mutations in downstream signaling molecules such as PIK3CA, truncation of the receptor to remove antibody binding, and masking of the receptor by mucins [12]. Recent work by us and others have also suggested that microenvironmental factors may also play a role in conferring resistance to HER2-targeted therapeutics [13, 14]. However, many of these mechanisms have not yet been validated in clinical trials, making their clinical significance unclear. Recently, studies have suggested that HER2+ breast cancers of the basal subtype may be less responsive to standard of care HER2-targeted therapy compared to non-basal HER2+ breast cancers [15], although others have not found such differences [16]. However, other studies have indicated that patients with the basal subtype may have poorer outcomes than luminal subtype HER2+ tumors [17, 18]. A better understanding of resistance mechanisms and the impact of intrinsic subtype are critically needed in order to allow the development of novel therapeutic approaches to overcome resistance.

We compared gene expression levels between lapatinib resistant and sensitive HER2+ breast cancer cell lines and identified significantly differentially expressed genes. We used an siRNA screen to examine the effects of knockdown of the top 25 differentially expressed genes to determine their effect on tumor growth and aggressiveness. These efforts led to the identification of INHBA, which appears to drive an aggressive tumor phenotype in basal subtype HER2+ cells and is associated with poor outcome in basal HER2+ breast tumors. Knockdown of INHBA led to decreased growth rates, increased sensitivity to lapatinib, and decreased invasiveness. These studies suggest that INHBA may be an attractive target for therapeutic intervention in the basal subtype of HER2+ breast cancers.

## Materials and methods

### Cell cultures and reagents

The 21MT-1 cell line was provided by Kornelia Polyak and Ruth Sager at the Dana-Farber Cancer Institute of Harvard, Cambridge, MA. JIMT1 was obtained from Leibniz Institute-DSMZ, Braunschweig, Germany. All other cell lines were purchased from American Type Culture Collection (ATCC), Manassas, VA. 21MT-1 cells were cultured in Dulbecco’s Modified Eagle’s Medium Nutrient Mixture-12 (DMEM/F12) supplemented with 5% horse serum, 20 ng/mL epidermal growth factor, 500 ng/mL hydrocortisone, 10ug/mL human insulin, and 100 ng/mL cholera toxin. JIMT1, MDAMB361, and UACC893 cells were cultured in DMEM supplemented with 10% Fetal Bovine Serum (FBS). SKBR3 cells were cultured in McCoy’s 5A medium with 10% FBS. HCC1419, HCC1569, and HCC1954 were cultured in RPMI 1640 supplemented with 10% FBS. All cells were incubated at 37 °C and 5% CO2 in a humidified incubator. All cell identities were validated by genotyping, and all cultures were tested regularly to ensure absence of mycoplasma as described by us previously [13].

### siRNA screening

Screens were performed as described previously [19]. Briefly, siRNAs were purchased from Qiagen and GE Dharmacon. The Dharmacon library included 20 tested genes and one positive control gene PLK (Serine/threonine-protein kinase gene). siRNA for each gene includes a pool of four fragments. The cells were seeded on 96-well plate. After 16–22 h the cells were transfected with siRNA at 20 nm of final concentration using Oligofectamine according to the manufacture’s instruction. At 72 h post-transfection the cell viability was measured using the Cell Titer-Glo assay (Promega). The mean of the cell number was calculated across the plate for all treatments and p-values were calculated using standard t-tests. Hits were deemed significant if they were less than or equal to 1.5 standard deviations below the mean and had a p-value less than 0.05.

### siRNA transfection and growth assessment

Two siRNA sequences targeting human inhibin beta A were designed by Qiagen. Oligonucleotide sequences were Hs_INHBA_6: 5’-AGGUCAACAUCUGCUGUAATT-3’; Hs_INHBA_4: 5’-CCAUGUCCAUGUUGUACUATT-3’. The AllStar negative control siRNA that does not target any known mammalian gene was used as a negative control. The day before transfection, 21MT-1, JIMT1, HCC1569, HCC1954, HCC1419, MDAMB361, or UACC893 cells were plated at 1.2 × 10^5^–2.0 × 10^5^ cells per 2 ml per well in six-well plates. Transfection was done using Lipofectamine RNAiMAX (Invitrogen) in Opti-MEM containing 20 nmol/L of siRNA or scramble RNA when the cells reached 60–80% confluence. siRNA-treated cells were harvested from six-well plates 24 h post-transfection and reseeded in 96-well plates. The cells were imaged in an IncuCyte® Live-Cell Analysis instrument and the cell growth curves were plotted based on the cell confluence data.

### Protein preparation and western blotting

Cells were washed twice with Dulbecco’s phosphate-buffered saline (DPBS) (1x) and lysed in radio immunoprecipitation assay (RIPA) buffer (Sigma) supplemented with Halt protease and phosphatase inhibitor cocktail (Thermo Scientific). Secreted INHBA proteins were precipitated from conditioned medium Opti-MEM using 10% (w/v) trichloroacetic acid. Protein concentration of the lysates was estimated using BCA protein assay kit (Pierce). The proteins were separated on 4–12% gradient Bis–Tris polyacrylamide gel (Invitrogen) and transferred onto a nitrocellulose membrane (GE Healthcare). The membrane was blocked by Odyssey® Blocking Buffer (Li-Cor), then incubated overnight at 4 °C with primary antibody (anti-β-actin antibody, Abcam, 1:1000; anti-INHBA, OriGene, 1:1000; anti-AKT, Cell Signaling Technology, 1:1000; Anti-phospho-AKT, Cell signaling Technology, 1:1000; anti-SMAD2, Cell Signaling Technology, 1:1000; or anti-phospho-SMAD2, Cell Signaling Technology, 1:1000). The secondary antibody used were Alexa Fluor 680 donkey anti-rabbit-IgG, IRDye 680RD donkey anti-mouse, and IRDye 800CW goat anti-rabbit. The immunoblots were imaged on the LI-COR Odyssey® 9120 Infrared Imaging system.

### Drug treatment

Cells were seeded in 96-well plates. Twenty-four-h post-seeding the cells were treated in randomized triplicate with nine doses of each compound in 1:5 (single drug treatment) or 1:2 (combination drug treatment) serial dilution as previously described [20, 21] or single dose indicated in figure legends. Drug responses for the initial experiments were measured as the GI50 value (dose required to inhibit growth by 50%) as previously described [20, 21]. Later experiments made use of the GR50 metric instead. The GR50 measurement is closely related to GI50 and was developed to permit more reproducible drug response metrics [22]. We utilized an online GR50 calculator to determine drug response for these cells [22]. Drug responses are relative to the untreated condition for each cell line tested. For the serial dilution drug treatment, the highest drug doses were: Lapatinib, 10 µM; A-83-01, 10 µM; Follistatin, 500 ng/ml. Cell proliferation was estimated using the Cell Titer-Glo® assay (Promega) or imaged in an IncuCyte® Live-Cell Analysis instrument. Dose–response curves were plotted based on the cell confluence data. Lapatinib was purchased from Selleckchem; Follistatin, Activin A, Activin B, Activin AB, and Inhibin A were purchased from R&D Systems. All reagents were reconstituted according to manufacturer’s instructions.

### 3D matrigel cultures

3D assays were performed using a previously described 3D on-top approach [23]. The siRNA transfected cells were seeded on 3D on-top Matrigel® coated plate well. After 24 h, replace the medium with medium plus the 500uM final concentration of lapatinib or the medium with DMSO as a control. Cells were imaged at 96 h using phase-contrast microscopy (Zeiss Observer A1), and cell quantity was assessed using absorbance measurements of Alamar blue stains according to the manufacture’s instruction (Invitrogen).

### RNA isolation and quantitative real time PCR

The total RNA from siRNA transfected cells was extracted using Qiagen RNeasy Mini Kit, according to manufacturer’s instruction. Total RNA was reverse transcribed to cDNA using Superscript™ Reverse Transcriptase III (Invitrogen) with the random primer (Invitrogen). Quantification of gene expression was performed by real-time quantitative PCR using SYBR Select Master Mix (Applied Biosystems) in a real-time PCR detection system (Applied Biosystems Quantstudio™ 7 Flex). B2M housekeeping gene was used as internal control. The relative quantification of gene expression was analyzed by the ΔΔCt quantification method. The target gene sequence for real-time PCR primers is listed in Table 1. All samples were analyzed in triplicate with error bars representing standard deviation.

**Table 1:** Sequences for primers used in the real-time PCR assays and guide RNA sequences and screening primer sequences for CRISPR experiments

Primers	Sequence (5′ to 3′)	Purpose
	AAGTCGGGGAGAACGGGTATG	RT-qPCR
INHBA-R	TCTTCCTGGCTGTTCCTGAC	RT-qPCR
B2M-F INHBA-F	TGCTGTCTCCATGTTTGATGT	RT-qPCR
B2M-R	TCTCTGCTCCCCACCTCTAAGT	RT-qPCR
sgRNA275 FOR	CACCGCGCGATCAGAAAGCTTCATG	Guide RNA for CRISPR
sgRNA275 REV	AAACCATGAAGCTTTCTGATCGCGC	Guide RNA for CRISPR
sgRNA95 FOR	CACCGCGCACAGGACGGACAGTCGG	Guide RNA for CRISPR
sgRNA95 REV	AAACCCGACTGTCCGTCCTGTGCGC	Guide RNA for CRISPR
g95-surveyor-F1	ACAGCCACAAACCTACAGCAC	Competition-based PCR
g95-surveyor-R1	TCCACATACCCGTTCTCCCC	Competition-based PCR
g95-surveyor-F2	CCCTTGCTTTGGCTGAGAGG	Competition-based PCR
g95-surveyor-R2	CAATGCCAGCACCAACCTGA	Competition-based PCR
g275-surveyor-F1	TCAGCCAGAGATGGTGGAGG	Competition-based PCR
g275-surveyor-R1	GTGTGACCCGCTGGGTTTAG	Competition-based PCR
g275-surveyor-F2	GATGCCCTTGCTTTGGCTGA	Competition-based PCR
g275-surveyor-R2	CAATGCCAGCACCAACCTGA	Competition-based PCR
g95-F-in	AGCGCGGCCCCCGACT	Competition-based PCR
g95-R-in	GCACAGGACGGACAGTCGG	Competition-based PCR
g275-F-in	GAACGCGATCAGAAAGCTTCATG	Competition-based PCR
g275-R-in	CCCGACTTTGCCCACATGAA	Competition-based PCR

### Oligomycin and deoxyglucose treatment

JIMT1 siRNA or scramble RNA transfected cells were seeded in 96-well plates. After 16–22 h, the cells were treated with different concentrations of 2-deoxyglucose (Tocris) or Oligomycin (Abcam) as described in the Figure legend. The cell numbers were measured spectrophotometrically by using the Cell Counting Kit-8 (Dojindo Molecular Technologies) at 450 nm. The growth comparison curve was plotted based on the comparison with non-treatment cells.

### Matrigel invasion assay

The transwell and invasion assays were performed using a Biocoat Growth Factor Reduced Matrigel® Invasion Chamber (Corning) according to the manufacturer’s instructions. In brief, the invasion chamber was rehydrated with serum-free medium at 37 °C in a 5% CO_2_ incubator for 2 h. After rehydration, siRNA or scramble RNA transfected cells were seeded at 5 × 10^4^ cells in 500 ul serum-free medium on the upper wells of the Transwell inserts. 0.75 ml medium with serum or without serum was added to the lower wells of the Transwell inserts. After 24-h incubation, non-invading cells on the up surface of membrane were removed and washed with PBS, the invading cells on the lower surface of membrane were fixed with methanol (100%) and nuclear stained with DAPI (0.5 ug/mL). The number of invading cells (DAPI stained nuclei) was counted under Zeiss Axio Observer A1 Inverted phase Contrast Fluorescence Microscopy. The cell number ratio between serum-free and serum was calculated. All assays were carried out in triplicate.

### INHBA genome editing in 21-MT1 cells

Genome editing in 21MT-1 cells was performed based on previously described Cas9-mediated strategy via the nonhomologous end joining (NHEJ) method [24]. An online CRISPR Design Tool (http://tools.genome-engineering.org) was used for designing guide RNA. Two single-guide RNA fragments were used for generating CRISPR mutants, respectively. The primers for guide RNAs are listed in Table 1. Briefly, phosphated SgRNA fragment was introduced into Cas9 plasmid pSpCas9(BB)-2A-puro (PX459) by BbsI site and insertion was confirmed by Sanger sequencing. Transfection of plasmids containing guide RNA (1ug) into 21MT-1 cells was done using lipofectamine 3000 (Thermo Fisher Scientific) and puromycin (1ug/mL) selection was started 24 h post-transfection. Five days post-transfection, the cells were detached, diluted, and reseeded in 96-well plates for single cell clone selection. A genome editing control was done without targeting (empty vector px459), using the same transfection method.

To identify the mutant clones, genomic DNA was isolated using QuickExtract™ DNA Extraction solution (Epicentre) according to manufacturer’s instructions. The mutants were screened using competition-based PCR strategy as described [25] with the PCR primers listed in Table 1. The INHBA indel mutations on 21-MT1-sg95(2A-11) and 21-MT1-sg275(2B-12) were PCR amplified, cloned, and verified by Sanger sequencing.

### RNAseq clustering and analysis of siRNA cells

We used existing RNAseq data for visualization of selected genes expressed in the various HER2+ cell lines. RNAseq data was median centered for display purposes. RNA was isolated from INHBA siRNA-treated or scramble control-treated basal 21-MT1, HCC1954, and JIMT1 cells using RNEasy columns (Qiagen). Luminal SKBR3 cells were used as controls. RNA was submitted to the OHSU massively parallel sequencing core facility for 50 base pair, paired-end sequencing. The RNAseq data was processed using the FPKM method. Since there were a small number of conditions we looked primarily at fold change and absolute change in expression levels between siRNA-treated and scramble control-treated cells in 21-MT1 and JIMT1, the two basal lines in which INHBA knockdown showed an impact.

### Electron microscopy

21-MT1 cells were treated with INHBA siRNA or scramble control for 48 h. The cells were washed once in 0.1 M sodium cacodylate buffer, pH 7.2, and then fixed in Karnovsky's solution (0.1 M Na cacodylate buffer containing 2% paraformaldehyde and 2.5% glutaraldehyde) for 30 min at room temperature, scraped into this solution, centrifuged into Eppendorf tubes, and stored at 4 °C before processing for EM. Following this fixation step, the samples we processed via microwave-assisted methods using a Pelco BioWave® Microwave. In the BioWave®, samples were rinsed in 0.1 M Na cacodylate buffer, incubated in reduced osmium tetroxide (1.5% w/v potassium ferrocyanide in 2% v/v OsO4), rinsed in water, and *en bloc* stained with aqueous 0.5% w/v uranyl acetate. Following the uranyl acetate incubation, samples were dehydrated in an aqueous series of 50% v/v, 75% v/v, and 95% v/v acetone, followed by two exchanges in 100% acetone. Epon resin infiltration was facilitated by incubation in a 1:1 solution of 100% acetone/freshly made Epon resin, followed by 4 exchanges in 100% freshly made Epon. Samples were removed from the BioWave® and transferred into embedding molds filled with freshly made Epon and cured at 60˚C for 36 h. Thin plastic sections (~ 70 nm) were obtained using a Leica UC7 ultramicrotome and imaged at 80 kV on a FEI-Tecnai T12 system interfaced to a digital camera and associated software (Advanced Microscopy Techniques, Danvers, MA).

### Statistical analysis

Standard statistical approaches were used in analyzing the data. We used Pearson correlation coefficients to compare pairs of genes, followed by standard t-statistics for significance of associations. We used t-tests for comparison of growth of cells in single endpoint assays. For comparison of growth curves, we used a permutation-based test known as CGGC (compare groups of growth curves), with 10,000 permutations to give robust p-value estimates, as described elsewhere [26]. Finally, we used Kaplan–Meier analysis for assessment of significant differences in survival rates using the GOBO database.

## Results

### Differential gene expression and siRNA screening identifies INHBA as a modulator of cell growth and lapatinib response in breast cancer cell lines

**Fig. 1:**
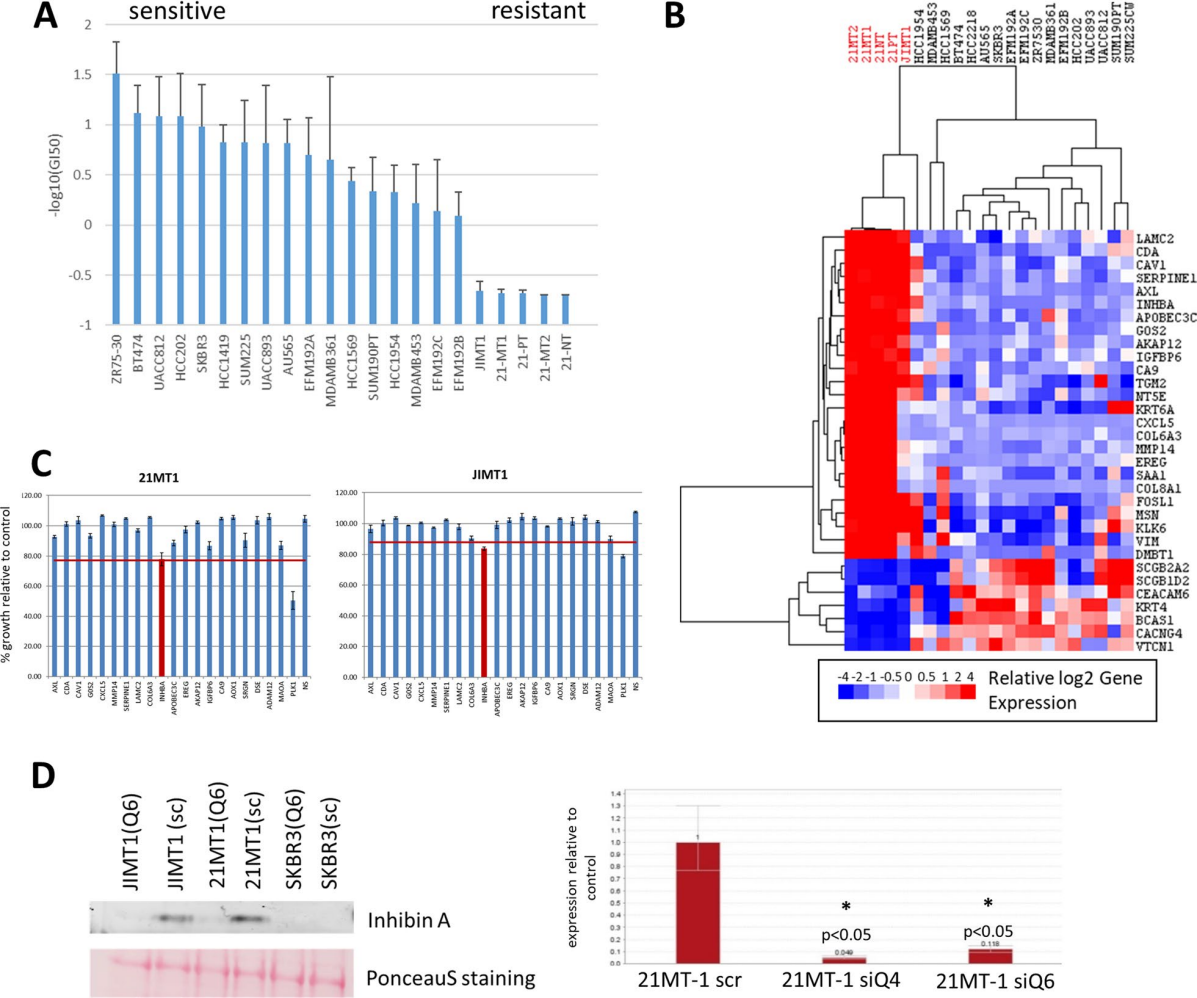
**A** Lapatinib response varies across 20 HER2^+^ breast cancer cell lines as measured by GI50 metrics. Cell lines to the right are more resistant, with the –log(GI50) set to the maximum dose tested. Error bars represent ± Standard Deviation (S.D.). **B** Heatmap of relative expression of genes that are differentially expressed by 20-fold or more between resistant and sensitive cell lines. The top 20 genes more highly expressed in resistant cells were selected for siRNA knockdown. **C** Cell growth relative to untreated control cells for JIMT1 and 21-MT1 cells treated with siRNAs against the top 20 genes from (**C**). Significant hits were deemed to be those that had cell viability at or below a value 1.5 S.D. from the mean, as described previously [19]. Error bars are ± S.D. **D** Analysis of protein (left panel) by Western blot and gene (right panel) expression by q-RT-PCR show that INHBA protein and transcript levels are decreased following siRNA knockdown. The knockdown of INHBA by siRNA Q4 results in 4.9% expression relative to control, while Q6 results in 11.8% expression

**Fig. 2:**
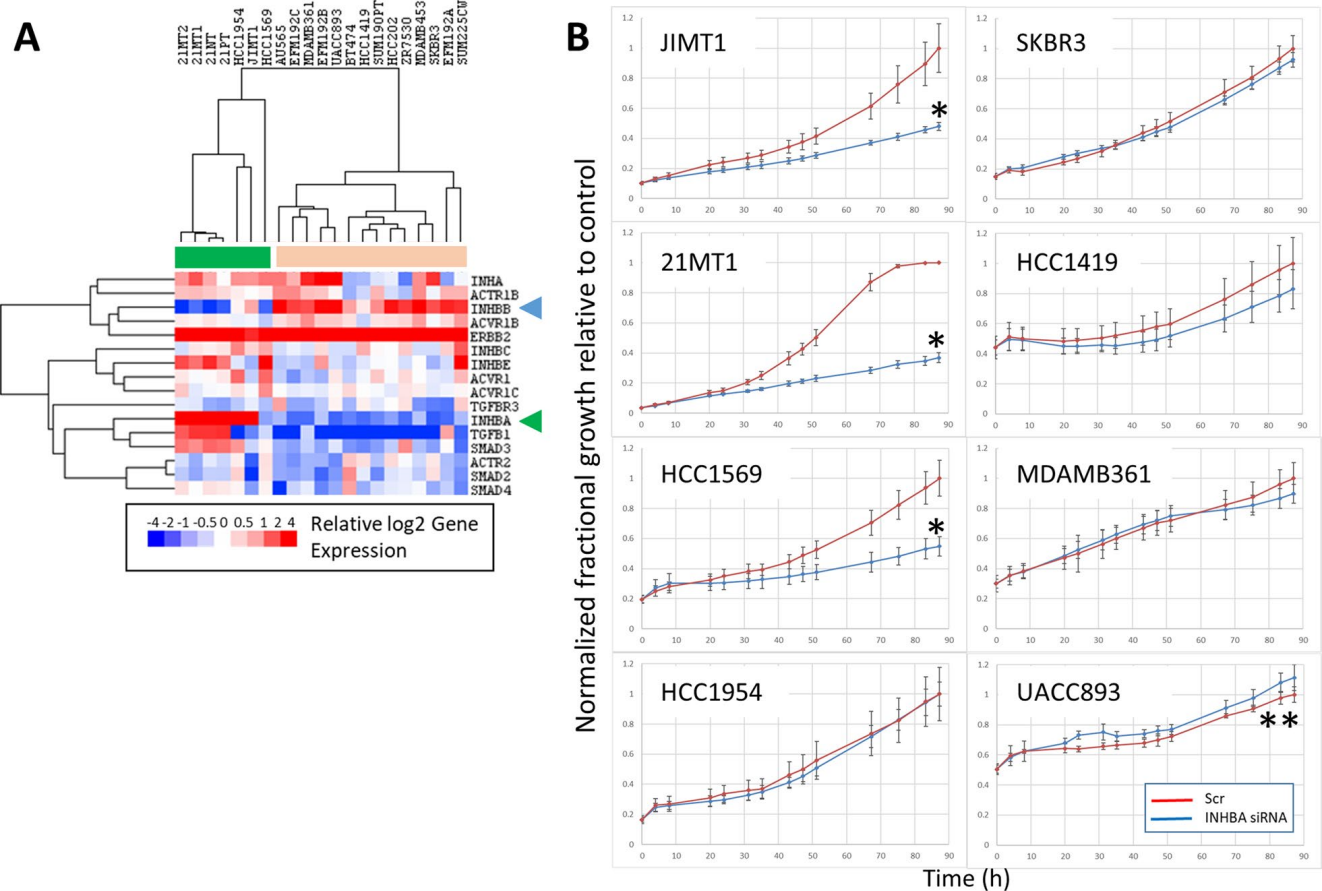
**A** Heatmap showing relative expression of INHBA and related genes in the HER2+ subset of breast cancer cell lines. Green bar indicates cluster containing basal cell lines, orange bar indicates cluster containing luminal cell lines. INHBA (blue arrow head) and INHBB (green arrow head) are inversely expressed in basal and luminal cells, respectively. **B** Normalized relative growth curves showing impact of knockdown of INHBA in four basal (left column) and four luminal (right column) HER2^+^ cell lines. Knockdown (blue) results in significant growth impairment in 3 out of 4 basal cell lines compared to scramble control-treated cells (red). In contrast, growth is not impacted by INHBA knockdown in any of the luminal cell lines, with the exception of UACC893, which saw a small increase in growth rate with knockdown. Cell numbers were normalized to account for different numbers of cells at the start of the assay, and values are relative to cell numbers of the control cells at the end of the assay. Error bars are ± S.D. Asterisks indicate significant differences between growth curves by permutation test; *p < 0.005; **p < 0.05

We utilized a large panel of molecularly characterized HER2+ breast cancer cells on which we have previously reported [20, 21, 27] to study lapatinib response. We performed dose response studies to determine GI50 values (dose required to inhibit growth by 50%) to identify HER2+ breast cancer cell lines that were sensitive or resistant to lapatinib treatment (Fig. 1A). We used RNAseq data that we had previously generated to identify genes that were differentially expressed between resistant (*N* = 5) and sensitive (*N* = 17) cell lines [20, 27]. This also corresponded to subtype status based on intrinsic subtyping previously reported by us [20, 21], as all the resistant lines belong to the basal intrinsic subtype, while 15/17 of the sensitive lines were of the luminal intrinsic subtype (all but HCC1954 and HCC1569). We found 25 genes that were more highly expressed by at least 20-fold in the resistant cell lines than in the sensitive cell lines at baseline (Fig. 1B). We obtained siRNA against the top 20 targets and assessed the effects of knockdown on the growth of 21-MT1 and JIMT1 (resistant) and SKBR3 (sensitive) cells at baseline and in the presence of 250 nM lapatinib. We found that knockdown of INHBA (which codes for the Inhibin βA subunit of Inhibin and Activin protein complexes) caused significant growth reduction compared to the other 20 siRNAs, and similar levels of growth inhibition as the PLK-targeted control siRNA (Fig. 1C) in 21-MT1 and JIMT1 cells. In contrast, knockdown of this gene did not impact growth of SKBR3 (Additional file 1: Fig. S1A). We repeated the knock down experiments of INHBA using two independent siRNAs, both of which also reduced the growth of 21-MT1 and JIMT1 cells (Additional file 1: Fig. S1B), increasing the likelihood that we are observing on-target effects. We could knock down INHBA with high efficiency and saw marked reduction of protein levels in JIMT1 and 21-MT1 cells (and RNA levels in 21-MT1 cells -JIMT1 cells were not tested) as shown in Fig. 1D, resulting in reduced proliferation (Figs. 1C, 2B).

We examined RNA expression of *INHBA* and related family members and signaling proteins in our breast cancer cell line panel. Examining expression in all breast cell lines (not just HER2+ cells), high levels of *INHBA* expression were limited mainly to basal subtype cells and the expression was largely inversely correlated with *INHBB* (*R* = − 0.42, *p* < 0.0005; see Additional file 1: Fig. S2A). In the HER2+ cell lines, we found that *INHBA* and *INHBB* expression was inversely correlated (*R* = − 0.76, *p* < 0.0001), with high levels of *INHBA* expression exclusively in basal HER2+ breast cells and high levels of *INHBB* expression limited mainly to luminal HER2+ cells (Fig. 2A). We also found expression of *INHA*, which forms the alpha subunit of functional Inhibin, in both the basal cells and a subset of the luminal cells (Fig. 2A). *INHBA* is a member of the TGFβ family of proteins that is known to signal through ACVRI receptors via SMAD2/3, so we also examined expression of related family members, receptors, and signaling molecules. *TGFβ1* (*R* = 0.70, *p* < 0.0005) and *SMAD3* (*R* = 0.70, *p* < 0.0005) were most significantly correlated with INHBA at the expression level. The expression of these genes was highest in the basal HER2+ cells, and largely absent in the luminal subtype cells (Fig. 2A). *SMAD4* showed a similar pattern of expression, although its expression was weaker and did not show as great a difference between the subtypes. None of the canonical TGFβ-SMAD genes showed a significant positive correlation with *INHBB* expression in the luminal subtype cells (Fig. 2A) except for *ACVR1B* (*R* = 0.50, *p* < 0.05).

Since there was a clear difference in *INHBA* expression between basal and luminal HER2+ cell lines, we expanded our knockdown testing to additional HER2+ cell lines to compare subtype effects of knockdown of INHBA. We tested four basal and four luminal subtype HER2+ cell lines that were treated with INHBA targeting siRNA. INHBA knockdown led to diminished growth relative to scramble control cells in 3 out of 4 basal lines (*p* < 0.005), while it did not inhibit growth in luminal lines (note that growth of UACC893 was slightly enhanced by knockdown of INHBA, *p* < 0.05; see Fig. 2B). Knockdown of *INHBA* with siRNA resulted in at least a twofold reduction in *INHBA* mRNA levels in these basal subtype HER2+ cell lines that highly expressed *INHBA* (Additional file 1: Fig. S2B).

**Fig. 3:**
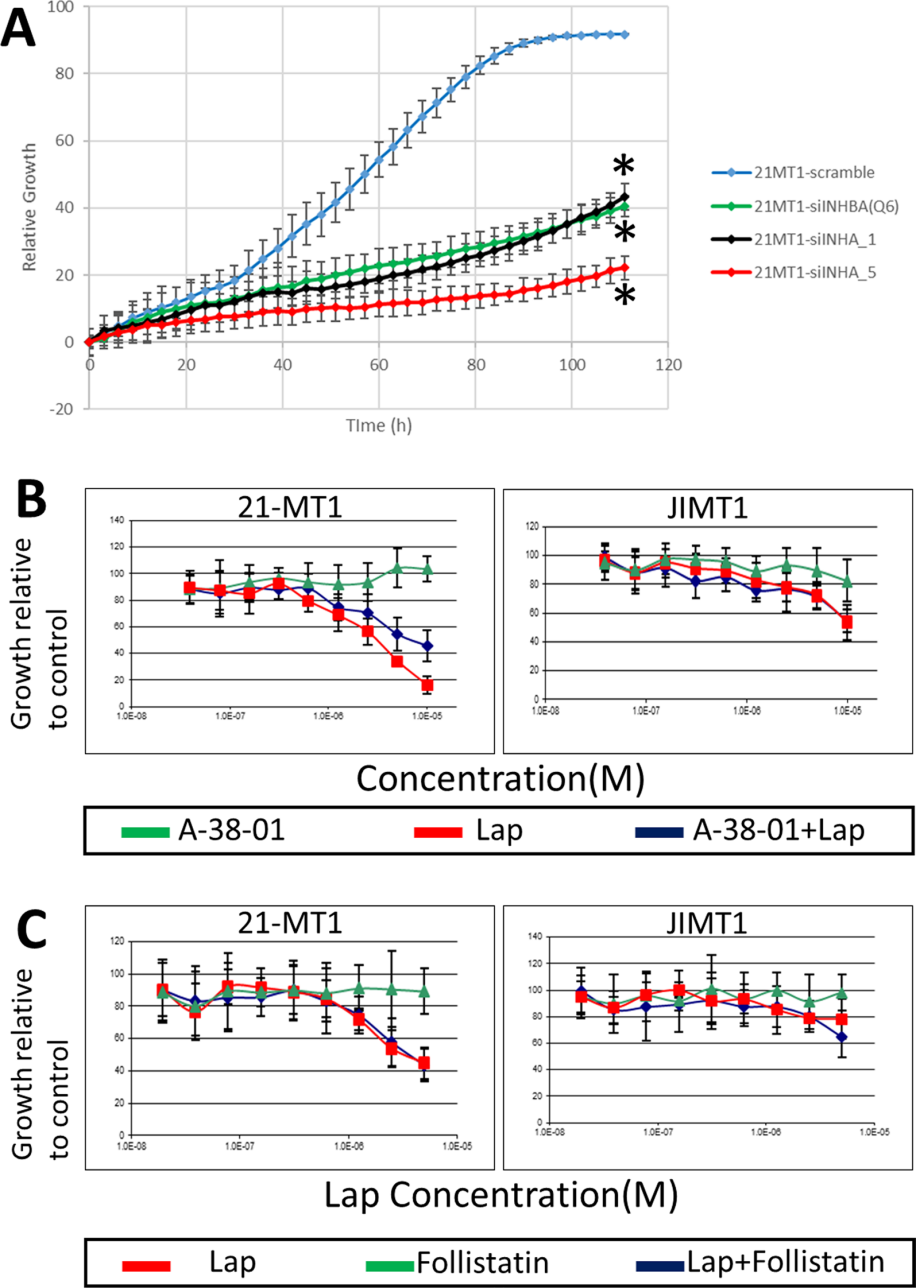
**A** Normalized relative growth curves showing impact of knockdown of INHBA and INHA in the basal HER2+ cell line 21-MT1. INHA and INHBA knockdown both result in significant growth inhibition (* indicates *p* < 0.001). **B** Treatment of 21-MT1 or JIMT1 cells with the ALK4/5/7 receptor inhibitor A-38-01 has minimal impact on cell growth. Combinations of A-38-01 plus lapatinib are ineffective compared to lapatinib alone (p-values comparing curves not significant for either JIMT1 or 21-MT1) and show evidence of antagonism at high concentrations in 21-MT1. **C.** Treatment of 21-MT1 or JIMT1 cells with follistatin, a competitive inhibitor of Activin, has no impact on cell growth (*p*-values comparing lapatinib to lapatinib plus follistatin response curves not significant for either JIMT1 or 21-MT1)

As mentioned above, INHBA codes for a subunit found in both Activin and Inhibin protein complexes, which canonically are secreted complexes that control the expression of follicle stimulating hormone (FSH), and also can impact expression of luteinizing hormone and production of androgens (Activins stimulate production of these hormones, while Inhibins block their production) [28–30]. INHBA can homodimerize with itself or heterodimerize with INHBB, resulting in complexes known as Activins, which activate signaling through ACVRI/ACVRII receptor dimers that are members of the TGFB signaling family [28, 29, 31]. In contrast, heterodimerization of INHBA with INHA results in formation of inhibin, which inhibits the activation of ACVRI/ACVRII receptors [28, 29, 31]. Since both INHBA and INHA are expressed in the basal subtype, it was unclear whether knockdown of INHBA was affecting Activin or Inhibin complexes (or neither). We treated 21-MT1 cells with siRNAs against INHA and found that knockdown of INHA could phenocopy INHBA knockdown (Fig. 3A), resulting in reduced proliferation. However, when we treated JIMT1 or 21-MT1 cells with the ACVR receptor inhibitor A-83-01 (Fig. 3B), we did not observe any growth inhibition, nor did it synergize with lapatinib to reduce proliferation of cells or improve lapatinib response (Fig. 3B). In fact, in 21-MT1 cells, addition of A-83-01 appeared to reduce the efficacy of lapatinib at high doses, suggesting that further inhibition of the receptor was detrimental to lapatinib therapeutic response. Similarly, addition of follistatin, which binds to and inhibits Activin [32, 33], had no impact on the growth of cells (Fig. 3C).

**Fig. 4:**
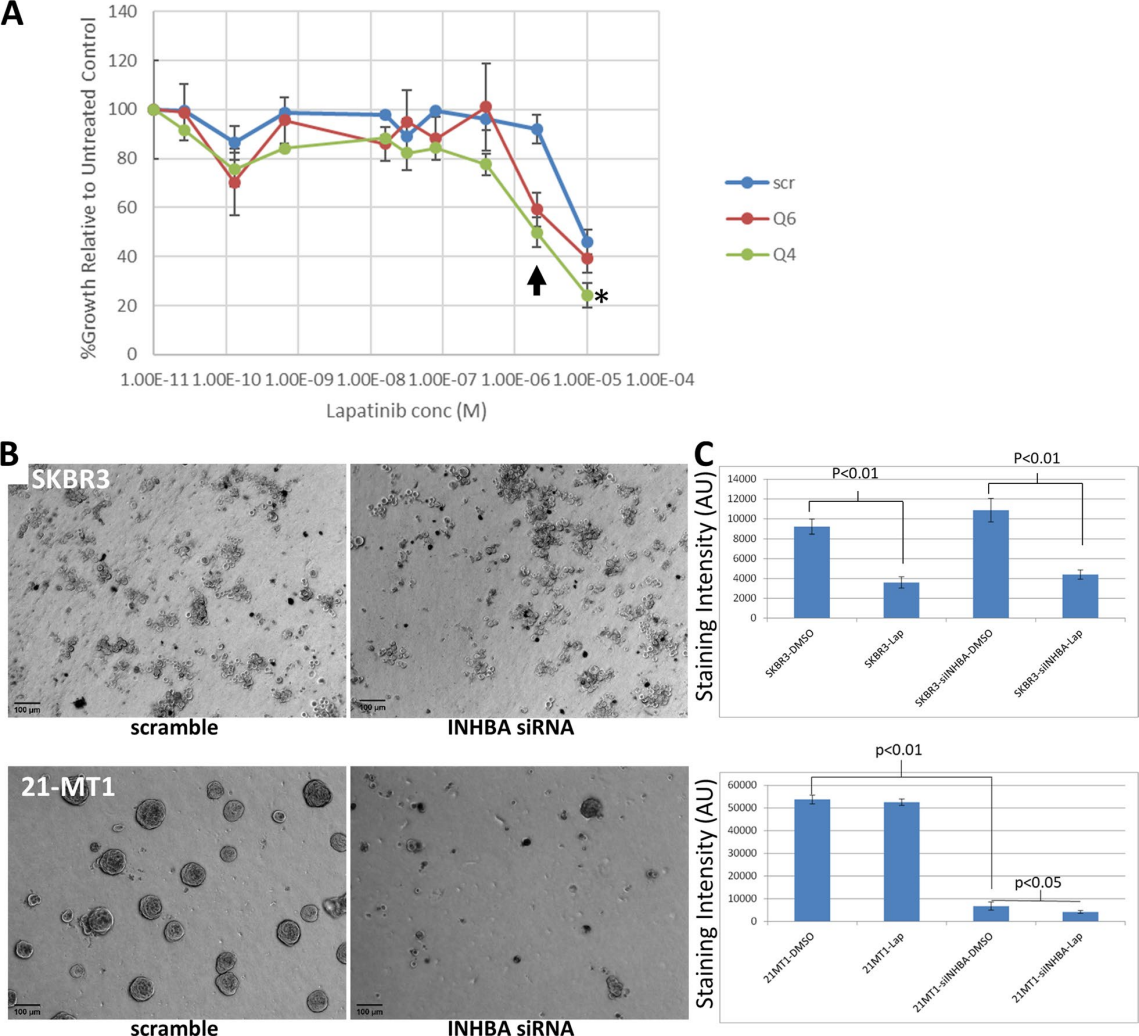
**A** Knockdown of INHBA sensitizes 21-MT1 cells to lapatinib as shown by % growth inhibition. Treatment with Q4 (green line) or Q6 (red line) siRNA resulted in both lower growth (not shown), as expected. Q4 siRNA increased sensitivity to lapatinib resulting in a left-shift of the curves compared to scramble-treated control (blue line; *p* < 0.001), while Q6 showed a trend but failed to reach significance (*p* = 0.16). In particular, note the differential sensitivity of the cells to 5 µM lapatinib (arrow). **B** Growth of 21-MT1 cells in 3-d Matrigel is inhibited by knockdown of INHBA, but has no effect on the growth of SKBR3 cells. **C** Quantification of growth and response of SKBR3 and 21-MT1 cells in 3-d Matrigel. INHBA knockdown significantly inhibits growth of 21-MT1 cells but lapatinib has minimal effect. However, 21-MT1 cells are more sensitive to lapatinib when INHBA is knocked down. In contrast, INHBA knockdown has no effect on the growth of SKBR3 cells, but lapatinib significantly impairs the growth of these cells. Error bars are ± Standard Error of the Mean (SEM)

We next tested the effect of INHBA knockdown on the response of 21-MT1 cells to lapatinib. While 21-MT1 cells treated with scramble siRNA controls were largely resistant to lapatinib, showing no response except at the very highest dose used (10 µM), cells treated with two different INHBA siRNA both showed marked decreases in proliferation at baseline as well as increased sensitivity to lapatinib, as evidenced by a left-shift of the dose response curves (Fig. 4A), although the difference was significant only for the Q4 siRNA. The GR50 (dose required to inhibit growth of the cells by 50%) decreased from ~ 10 uM in the scramble controls to 1 uM and 500 nM in the cells treated with the two different INHBA siRNAs in 21-MT1 cells. We next determined the impact of knockdown of INHBA on the growth and lapatinib response of HER2+ cells in 3-d cultures using the Matrigel on top method [23]. We grew 21-MT1 and SKBR3 cells (each transfected with either a scramble control siRNA or INHBA siRNA) in Matrigel, and treated them either with DMSO (vehicle control) or 500 nM lapatinib. As expected, the INHBA siRNA had no effect on the growth of luminal SKBR3 cells (Fig. 4B) and lapatinib treatment led to significant inhibition of growth of SKBR3 in Matrigel in both scramble and INHBA siRNA-treated cells (Fig. 4B). In contrast, the 21-MT1 cells treated with the scramble control siRNA were resistant to lapatinib, as there was no discernable difference in the number of cells that grew in Matrigel in the presence or absence of lapatinib (Fig. 4C). However, introduction of the INHBA siRNA resulted in both a reduction in the number of colonies that formed as well as the size of the colonies, manifested as a significant decrease in cell numbers (Fig. 4C). Furthermore, the INHBA siRNA-treated 21-MT1 cells were more responsive to lapatinib (Fig. 4C), resulting in significantly fewer cells than cultures treated with vehicle, consistent with the observations made with 2-d culture approaches.

### RNAseq reveals a potential metabolic switch in cells treated with INHBA siRNA

We next performed expression profiling by RNAseq on 21-MT1, JIMT1, and HCC1954 basal HER2+ cells or SKBR3 luminal HER2+ cells. All cells were treated with scramble control or INHBA siRNA in order to identify genes that were affected by loss of INHBA. We previously found that knockdown of INHBA had limited effects on SKBR3 and HCC1954 and so these were used primarily as controls for non-specific effects of treatment. We compared the changes in expression of 21-MT1 and JIMT1 cells with INHBA knockdown to the same cells treated with scramble control. We compared both the absolute change in expression as well as fold-change in expression to identify potential target genes. We first looked at genes with a large fold-change in expression. As expected, *INHBA* was down-regulated in the knockdown cells (Additional file 1: Fig. S2B). This also included the control SKBR3 cells, which showed an almost 15-fold reduction in expression, even though at baseline the expression level was ~ 100 times lower than the basal cell lines. This result is consistent with a strong on-target knockdown of INHBA with the siRNA.

**Fig. 5:**
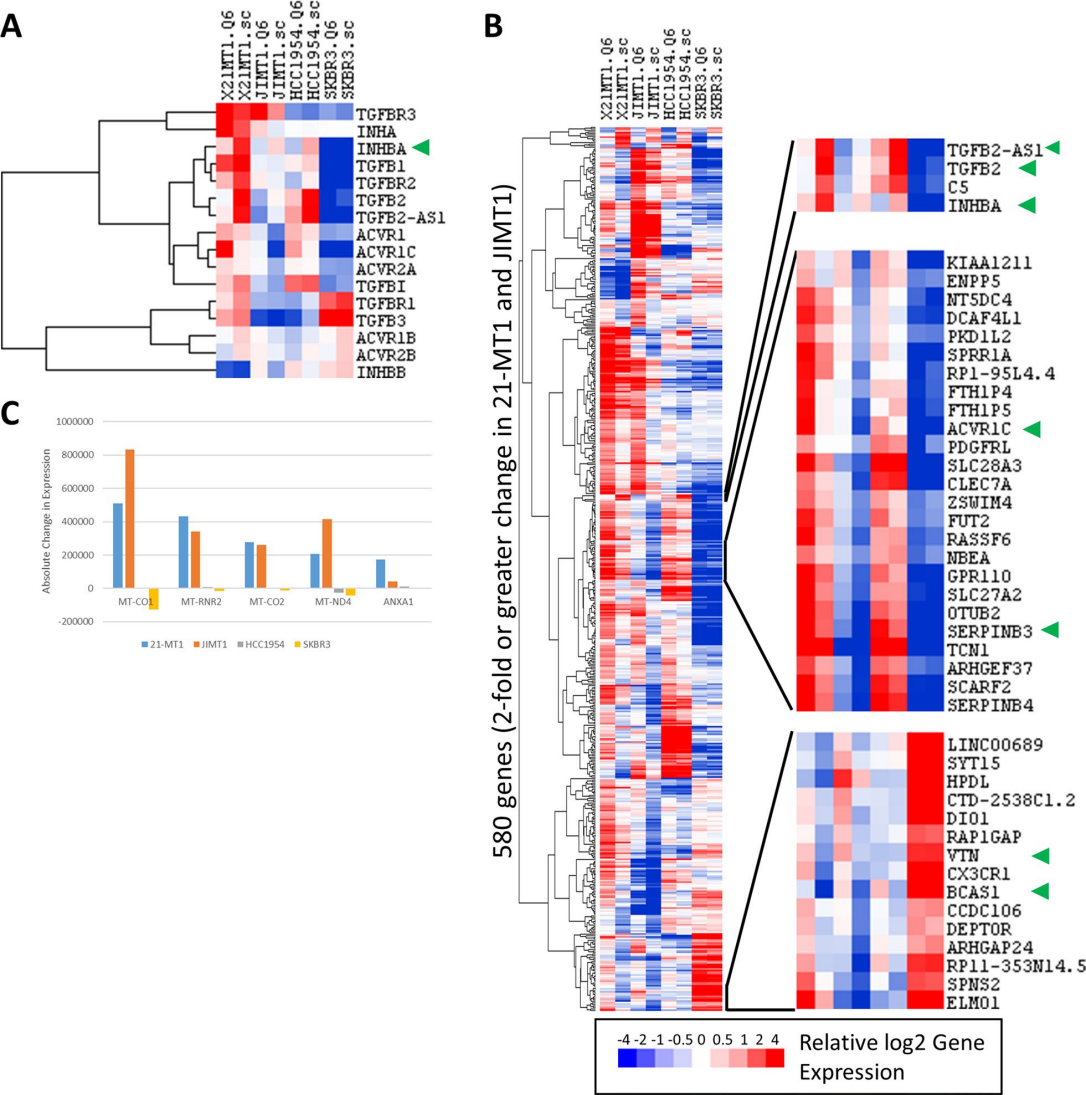
**A** Heatmap of gene expression of selected TGF related genes. INHBA is marked with an arrowhead. **B** Heatmap of genes that show the largest fold-change between siRNA-treated and control-treated cells. Genes highlighted in text are indicated with arrowheads. **C** Expression of genes that show the highest absolute change in expression between knockout and scramble control-treated 21-MT1 and JIMT1 cells. Graph shows absolute change in expression in siRNA-treated vs. scramble-treated controls. Mitochondrial genes are upregulated in both 21-MT1 and JIMT1 cells

We first looked at *IL6* and *IL13Rα2*, which have previously been reported to be regulated by INHBA in ovarian and breast cancers [34]. Surprisingly, we found that expression of these genes was not consistently altered by INHBA knockdown in any of the three basal subtype cell lines. *IL6* expression decreased twofold in INHBA siRNA-treated 21-MT1 cells, but increased by twofold in JIMT1 cells treated with the same siRNA. *IL6* expression also increased, but by less than twofold, in the HCC1954 basal cells treated with INHBA siRNA. Similarly, *IL13RA2* showed disparate expression between 21-MT1 and JIMT1 cells treated with INHBA siRNA, but in neither case was the expression of *IL13RA2* changed by twofold or more. HCC1954 cells did not show appreciable levels of expression of *IL13RA2* even at baseline. We next looked at genes that are associated with TGFβ signaling and activity (Fig. 5A). In addition to *INHBA* showing strong down-regulation, we saw that *TGFB2* and *TGFB-AS1* also showed significant and consistent decreases in expression in both JIMT1 and 21-MT1 cells. *ACVR1C*, one of the receptors for Activin, was up-regulated in response to INHBA knockdown in both 21-MT1 and JIMT1 cells. No other TGF-related genes showed consistent changes in gene expression.

In addition we looked at genes that showed large-fold or absolute change in expression. For fold-change, we looked at genes that were changed by at least twofold in both JIMT1 and 21-MT1 cells. We found 29 genes that were down-regulated and 551 genes that were up-regulated in response to INHBA knockdown. The most prominent genes that were down-regulated were again *INHBA*, *TFGB2*, and *TGFB2-AS1* (Fig. 5B). *SERPINB3*, *BCAS*, and *VTN* were among the genes that were most upregulated (Fig. 5B). For absolute changes in gene expression, we saw a large number of mitochondrial genes involved in oxidative phosphorylation upregulated in cells treated with INHBA siRNA (Fig. 5C). Concomitant with upregulation of mitochondrial genes, we saw down-regulation of *LDHA*, a central protein in glycolytic metabolism (albeit only in 21-MT1).

**Fig. 6:**
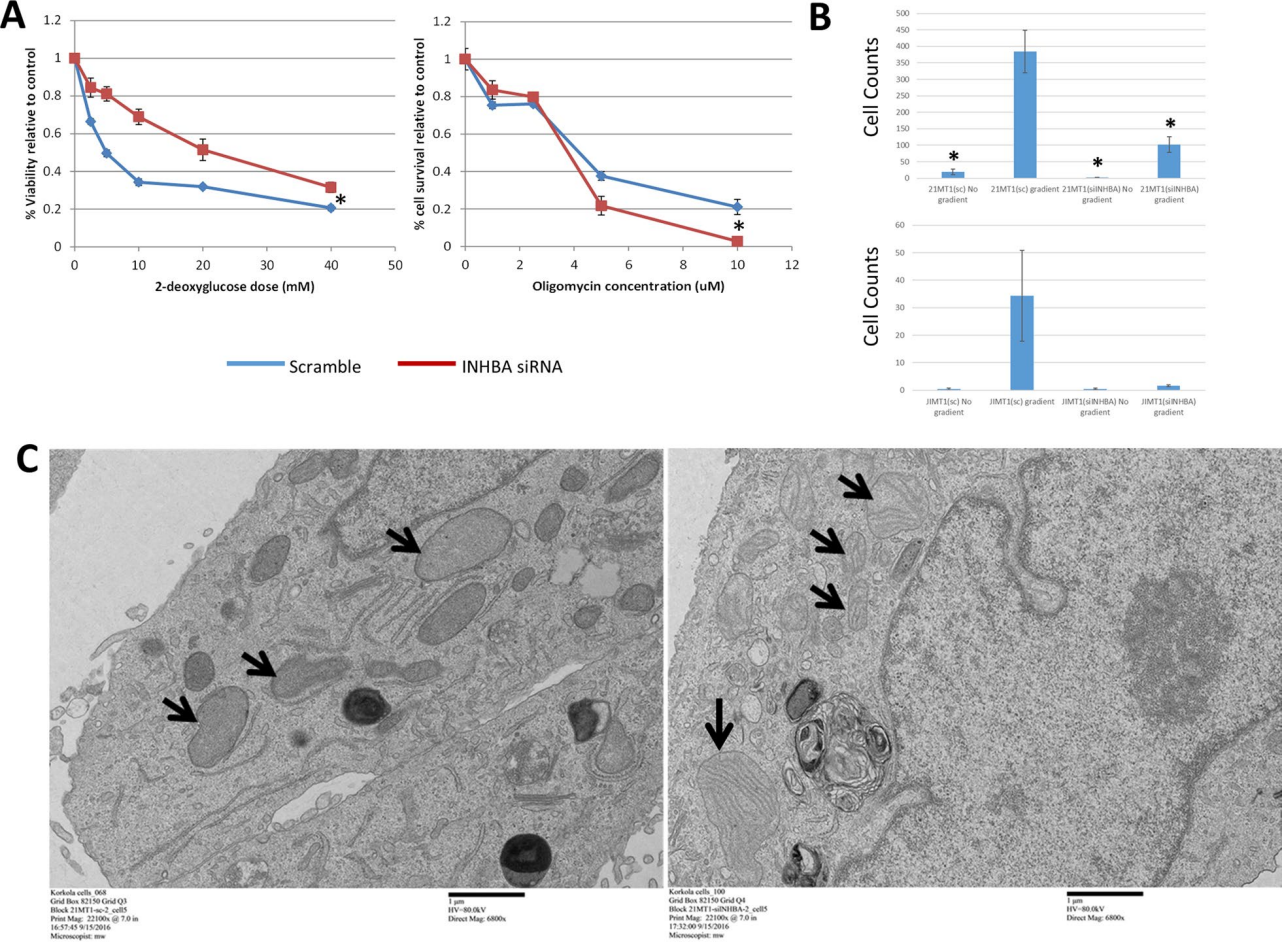
**A** Drug response of scramble or INHBA siRNA-treated 21-MT1 cells to the glycolysis inhibitor 2-deoxy-glucose or the oxidative phosphorylation inhibitor oligomycin shows that INHBA knockdown cells are more sensitive to oligomycin and less sensitive to 2-deoxy-glucose, consistent with a shift in metabolism away from glycolysis toward oxidative phosphorylation. Error bars are ± S.D. * indicates *p* < 0.005. **B** Invasion assays show that both 21-MT1 and JIMT1 wild-type cells are highly invasive through basement membrane toward media containing serum. In contrast, when INHBA is knocked down, the cells become much less invasive. * indicates significantly lower growth compared to scramble-treated cells migrating toward a gradient (*p* < 0.05 by Student’s t-test); error bars are ± S.D. **C** TEM images of 21-MT1 cells show a mixture of normal and abnormal mitochondrial structures (including the lack of cristae) in scramble-treated cells, in contrast to INHBA knockdown cells, which show distinct cristae present in the cells

The gene expression changes are consistent with a potential metabolic shift from glycolytic to oxidative phosphorylation metabolism. To test this, we treated 21-MT1 cells with either the scramble siRNA control or the INHBA siRNA for 48 h, then treated the cells with either 2-deoxyglucose (a glycolysis inhibitor) or oligomycin (an inhibitor of the electron transport chain in oxidative phosphorylation). We found that the INHBA siRNA-treated cells were less sensitive to 2-deoxyglucose but more sensitive to oligomycin than the scramble control-treated cells, consistent with a shift in metabolism from glycolysis to oxidative phosphorylation in these cells (Fig. 6A). It has previously been reported that inhibiting LDHA results in a shift from glycolysis toward oxidative phosphorylation in cancer cells and that this shift is associated with a loss of an aggressive phenotype, including reduced proliferation and invasion [35]. We had already demonstrated the loss of proliferative capacity and down-regulation of *LDHA* as a result of INHBA knockdown in 21-MT1 cells, but wondered whether loss of INHBA also influenced the invasive potential of these cells. To determine the effects of INHBA knockdown on invasive potential, we plated 21-MT1 and JIMT1 cells into Matrigel-coated membranes to assess their ability to invade toward a gradient (full serum). 21-MT1 scramble control-treated cells showed significant invasive potential toward the gradient, but no invasion if the gradient was absent (Fig. 6B). In contrast, the INHBA siRNA-treated 21-MT1 cells showed limited ability to migrate toward the gradient, with significantly fewer cells present compared to control. JIMT1 cells showed the same pattern of response, although the differences failed to achieve significance (*p* =  ~ 0.10).

We also performed transmission electron microscopy (TEM) on a small number of 21-MT1 cells treated with scramble or INHBA siRNA (Fig. 6C: *N* = 5 for both). Qualitatively, we noticed a change in the rough endoplasmic reticulum (RER) of some of the siRNA-treated cells compared to normal, as the cisternae of the RER took on a more bloated appearance compared to the thin cisternae of the scramble-treated cells. Furthermore, in some of the cells, we noted a difference in the ultrastructure of the mitochondria. In the wild-type (scramble-treated) cells, some of the cells had mitochondria that lacked noticeable cristae, or had few cristae (Fig. 6C). In contrast, in the INHBA siRNA-treated cells, there were many more cristae evident. This observation is interesting, given the effects we previously had seen on metabolism, but we gathered too few images to make definitive conclusions about the effects of knockdown on mitochondrial structure in these cells.

### Exogenous activin or inhibin protein does not rescue INHBA siRNA treatment

**Fig. 7:**
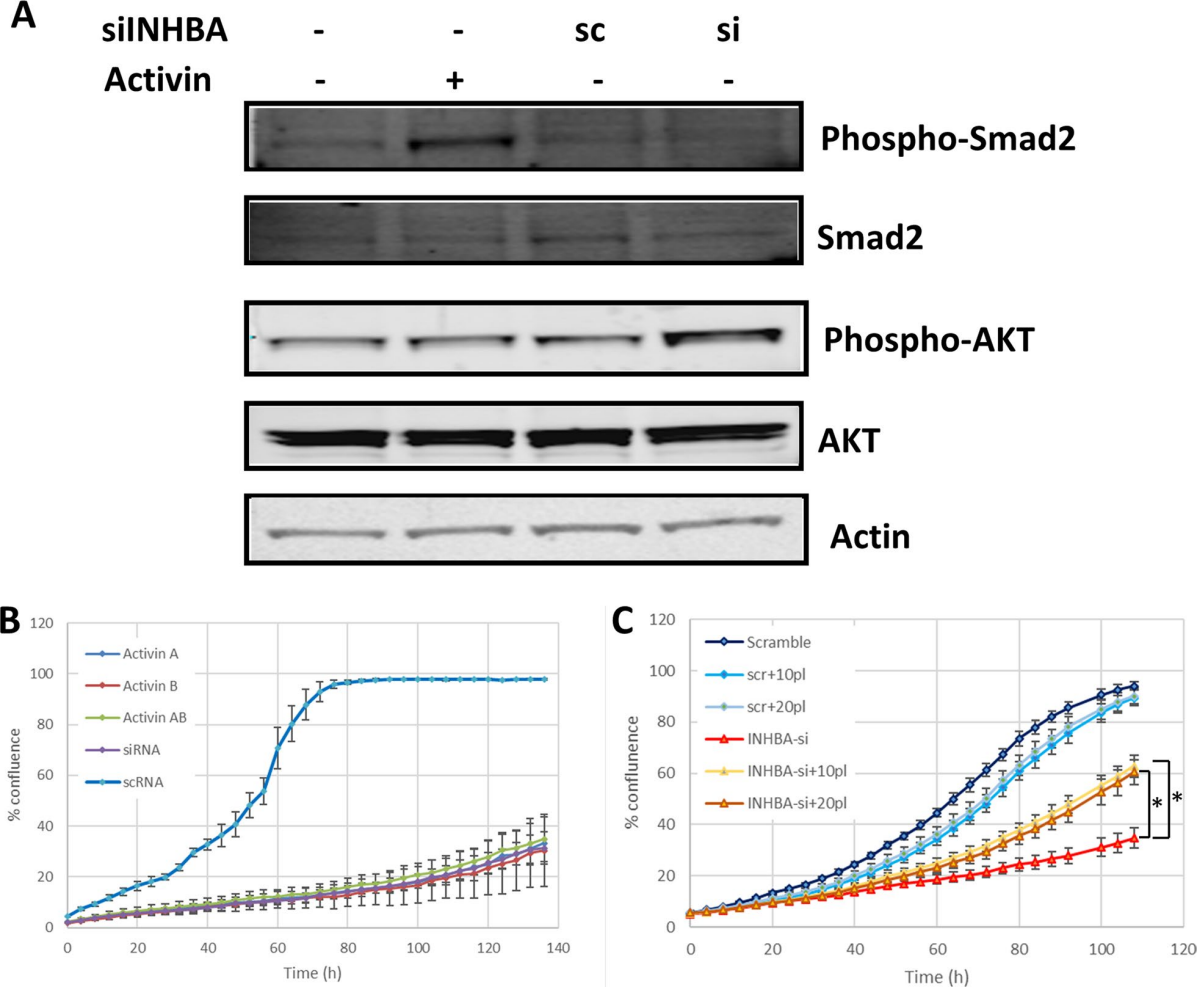
**A** Western blot showing effects of treatment of 21-MT1 cells with 10 ng/ml recombinant Activin or siRNA against INHBA. Activin treatment activates p-SMAD2 in 21-MT1 cells. Knockdown of INHBA has minimal impact on the p-SMAD2 levels compared to baseline conditions. **B** Growth curves for 21-MT1 cells treated with different types of recombinant Activin or Inhibin. Cells were treated with siRNA against INHBA then 50 ng/ml recombinant protein was added to the siRNA-treated cells and growth was assessed for 144 h. No Activin+ siRNA-treated cells showed any significant growth rescue compared to siRNA alone treated cells. Error bars are ± S.D. **C** Growth curves for 21-MT1 cells treated with scramble control siRNA or INHBA siRNA plus 10 or 20 ng/ml of an siRNA-resistant INHBA cDNA plasmid. Addition of 10 or 20 ng/ml plasmid results in significant rescue of the growth inhibition caused by INHBA knockdown (*p* < 0.001). Error bars are ± S.D

Our data found that inhibition of the receptor and addition of competitive protein complexes did not impact growth of basal subtype cells that had high levels of INHBA expression (Fig. 3). We next sought to understand if exogenous Activin or Inhibin protein could rescue the reduction in growth caused by silencing of INHBA by siRNA. We treated 21-MT1 cells with recombinant Activin AB and saw that it was functional, as we observed upregulation of p-SMAD2 (Fig. 7A). However, when the protein was added to INHBA siRNA-treated 21-MT1 cells, we saw no rescue of the growth inhibition (Fig. 7B). Similarly, other Activin and Inhibin proteins were unable to rescue the growth defect caused by INHBA knockout (Fig. 7B). These data suggested that INHBA might be acting in a non-canonical manner to drive basal HER2+ breast tumor growth and aggressiveness. We made a cDNA plasmid that contained an INHBA cDNA that had mutations in the third position of the codons that were targeted by our siRNA, and thus the construct would code for the same protein sequence as the endogenous gene, but is not efficiently targeted by our siRNA. When this was transfected back into 21-MT1 cells, the siRNA no longer inhibited the growth of the cells (Fig. 7C). This supports the hypothesis that INHBA might be functioning through a different mechanism than the canonical receptor signaling pathway.

### CRISPR knockout of INHBA

**Fig. 8:**
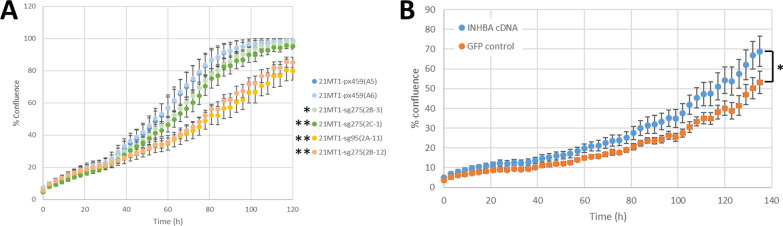
**A** Growth curves for 21-MT1 cells with INHBA deleted by CRISPR targeting. Px459 cells went through the selection process but no guide RNA was added and serves as a control. The sg275(2B-12) and sg95(2A-11) CRISPR-treated clones both showed frameshift mutations that altered the coding sequence of the gene. The sg275(2B-3) and sg275(2C-1) CRISPR-treated clones showed in-frame deletions of 2 amino acids, resulting in maintenance of one copy of the protein sequence. Examination of growth rates showed that the clones with effective CRISPR knockout of INHBA grew significantly slower than the controls, as did in-frame CRISPR mutants. Error bars are ± S.D. (* indicates *p* < 0.05 compared to px459(A5) controls; ** indicates *p* < 0.001 compared to px459(A5) controls). **B** Treatment of 21-MT1 2A11 clone with 100 ng of an INHBA cDNA increases growth rescue compared to GFP control plasmid-treated cells (*p* < 0.001)

Since siRNA can be non-specific and does not knock down 100% of protein, we turned to CRISPR as a final validation of the impact of INHBA loss in basal HER2+ cells. We prepared constructs that had two different guide RNAs targeting INHBA (sg275 and sg95). We treated 21-MT1 cells with the guide RNAs or control (px459) and then isolated single clones for sequencing to verify knockout. We identified two clones that had effective CRISPR deletion that resulted in frameshift of the coding sequence and thus altered the protein (sg275 clone 2B-12 and sg95 clones 2A-11). We also found two clones that had frameshift that deleted two amino acids but left the protein otherwise intact (sg275 clone 2B-3 and sg275 clone 2C-1). We tested the growth of these and the px459 treated control cells and found that the clones with frameshift knockouts resulted in impaired growth compared to both the control cells and the in-frame clones (Fig. 8A). Introduction of a plasmid expressing INHBA partially restored growth to these cells, compared to a GFP control plasmid (Fig. 8B; also compare with Fig. 8A).

### INHBA expression in patient tumors and clinical implications

**Fig. 9:**
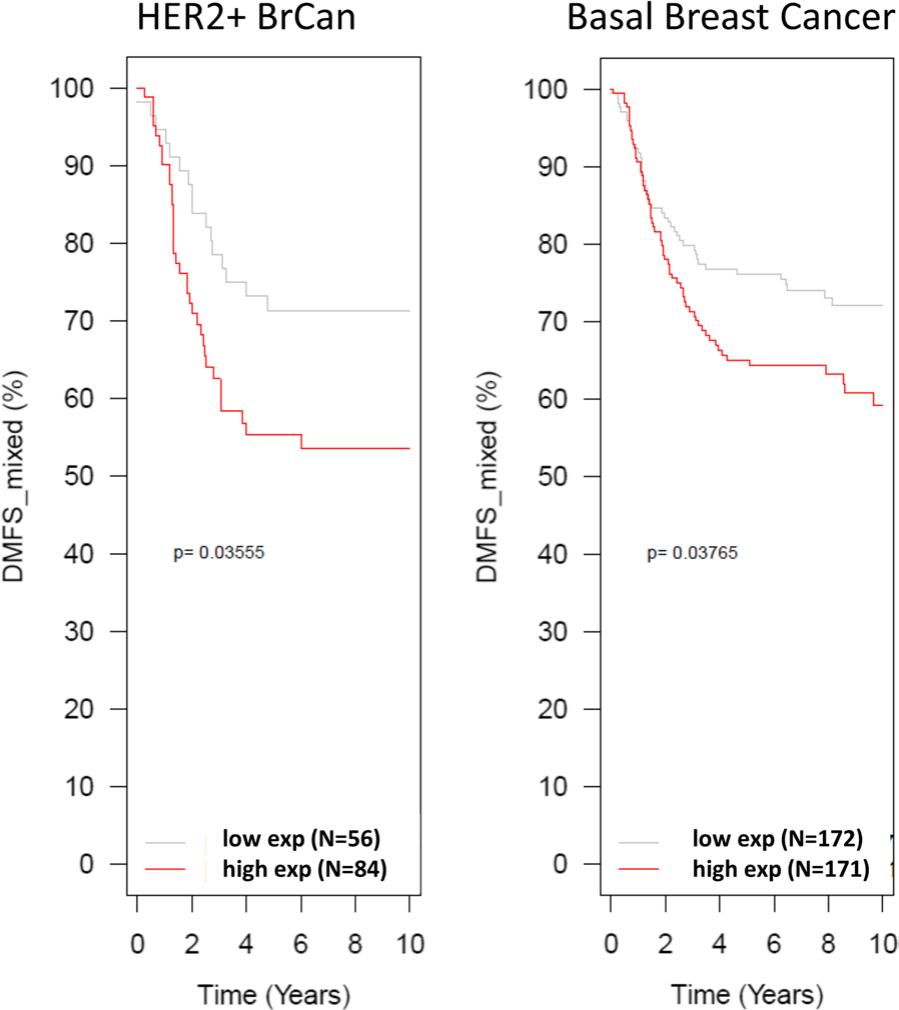
Kaplan–Meier survival curves of HER2+ and basal subtype breast cancer cells shows that high levels of INHBA is associated with poor outcome in these patient subgroups. In contrast, there is no difference in survival based on INHBA levels in luminal subtype breast cancer patients (see Additional file 1: Fig. S3B)

We examined expression of INHBA in large public datasets to determine the impact of high levels of expression on outcome in breast cancer. We examined outcome using GOBO [36], which compiles large numbers of breast expression datasets for gene analysis with outcome. Here, we found that INHBA showed both variability in expression and high levels of expression as expected (Additional file 1: Fig. S3A). Both basal subtype tumors and HER2+ tumors showed significant associations between high levels of INHBA expression and poor outcome (Fig. 9). In contrast, luminal tumors showed no association with outcome, as expected from our in vitro data (Additional file 1: Fig. S3B).

## Discussion

Although treatment advances for patients with HER2+ breast cancer have significantly improved outcomes, treatments are still suboptimal for patients with advanced metastatic disease [12]. This is largely due to resistance to therapy. We looked at differences in gene expression between HER2+ cell lines that are resistant and lines sensitive to HER2-targeted inhibitors, then performed an siRNA screen on genes that were more highly expressed in resistant lines. This approach identified *INHBA* as a gene that regulates cell proliferation, invasion, and therapeutic response in the basal subtype of HER2+ breast cells. *INHBA* codes for a protein that is found as a subunit in both Activin and Inhibin protein complexes that are involved in the regulation of FSH and luteinizing hormone [28–30]. *INHBA* has been shown to be associated with poor outcome in a variety of tumors, including breast [34, 37], lung [38, 39], and bladder [40]. Previous studies in the breast had shown that Activin A regulates breast tumor aggressiveness through IL13Ra2 to promote metastatic spread, and that this occurs primarily in basal-like breast cancers [34]. In our studies, we did not see any association between IL13Ra2 and INHBA expression, perhaps due to the fact that we focused on HER2+ cells in our study compared to non-HER2 basal cells in the previous study [34]. While many of our observation were consistent with the work of Kalli et al., such as the effects of knockdown of INHBA delaying cell growth and reducing migration, we did not find that inhibition of the receptor could inhibit the growth of cells. Again, this difference may reflect fundamental differences between basal and HER2+ breast cancer cells. Others have also shown that Activin may alter fibroblasts in the local tumor microenvironment to secrete pro-tumorigenic factors, resulting in increased malignancy and invasiveness of the tumor cells [41]. Thus, *INHBA* may have additional indirect effects that promote tumor growth beyond those that we have observed in our cell line studies.

Knockout of INHBA reduced cell proliferation, decreased invasiveness, and increased sensitivity to lapatinib in our studies. Knockdown of INHBA altered metabolism, with an apparent shift from glycolytic to oxidative phosphorylation, and also altered the RER structure and possibly mitochondria structure in some of the siRNA-treated cells. Interestingly, previous studies have indicated that inhibition of *LDHA*, which we observed was decreased following INHBA knockdown, results in decreased proliferation and impaired tumor invasion and progression [35, 42], providing an additional link between INHBA, the loss of proliferation and invasion we observed, and changes in metabolism. However, further studies are required to definitively determine the impact of INHBA knockdown on metabolism, and whether this is a direct effect or merely a consequence of the decreased proliferation.

Our findings suggest that INHBA plays a central role in conferring an aggressive phenotype to basal subtype HER2+ breast tumors. However, the mechanism by which INHBA is acting remains unclear. Inhibition of the canonical ALK receptors for Activin, of which INHBA is a subunit, had no effect on the growth of cells. Similarly, treatment with follistatin, which binds to Activin and blocks its activation of ALK receptors, had no impact on the growth of cells. When we knocked down *INHBA* and added back either Activin or Inhibin, we were unable to rescue the growth of cells. However, when we added back an siRNA-resistant cDNA expressing INHBA, growth was largely restored. These data suggest that *INHBA* may be acting through a non-canonical pathway. Elucidation of the mechanism by which *INHBA* is acting remains an active area of study in our laboratory. Similarly, we observed that many non-HER2 basal subtype cells also expressed high levels of *INHBA*, and it will be interesting to determine if it plays a role in these tumors as well.

## Conclusions

Our data suggest that targeting *INHBA* may be a viable strategy to reduce invasiveness and improve growth control by HER2-targeted agents in the basal subset of HER2+ tumors. Although the effect of subtype remains unclear in HER2+ disease, there are indications that the basal subtype may be more aggressive and have worse outcomes than their luminal-like counterparts [15, 17, 18]. Targeting *INHBA* may not be straightforward, however, since our data suggests that simply inhibiting the receptor for Activin has no effect on the growth of these cells. Furthermore, Activin plays an important role in additional function including regulation of hormones and reproduction, erythropoiesis, nervous system function, and immune activity [29], so systemic inhibition may have serious side effects. Thus, we are investigating the possible use of nanoparticles [43] to perform targeted inhibition of *INHBA* as a therapeutic strategy. Elucidation of the mechanism of action of *INHBA* in HER2+ basal breast tumors will also help to devise optimal strategies for potential therapeutic inhibition of INHBA to improve outcomes in patients with advanced basal subtype HER2+ breast cancer.

## Supplementary Information

**Additional file 1: Figure S1A**. Knockdown of the top 20 selected genes in the sensitive cell line SKBR3 reveals no role for INHBA in the growth of these cells. B. Use of additional siRNAs against INHBA demonstrates growth inhibition in 21-MT1 cells. **Figure S2A**. RNAseq heatmap of INHBA and related protein expression in a panel of breast cancer cell lines. B. Quantification of the effects of knockdown of INHBA in basal and luminal HER2+ cell lines. **Figure S3**. Outcome in patients with luminal HER2+ breast tumors as a function of INHBA expression levels shows no significant association with survival.

## Data Availability

The datasets used and/or analyzed during the current study are available from the corresponding author on reasonable request.
