# Supplementary material for: INHBA is a mediator of aggressive tumor behavior in HER2+ basal breast cancer
Source: Breast Cancer Res. 2022 Mar 5;24:18. doi: 10.1186/s13058-022-01512-4 (PMC8898494; doi:10.1186/s13058-022-01512-4)
Supplement: Supplementary file 1 — Additional file 1: Figure S1A. Knockdown of the top 20 selected genes in the sensitive cell line SKBR3 reveals no role for INHBA in the growth of these cells. B. Use of additional siRNAs against INHBA demonstrates growth inhibition in 21-MT1 cells. Figure S2A. RNAseq heatmap of INHBA and related protein expression in a panel of breast cancer cell lines. B. Quantification of the effects of knockdown of INHBA in basal and luminal HER2+ cell lines. Figure S3. Outcome in patients with luminal HER2+ breast tumors as a function of INHBA expression levels shows no significant association with survival. [file 13058_2022_1512_MOESM1_ESM.pdf]

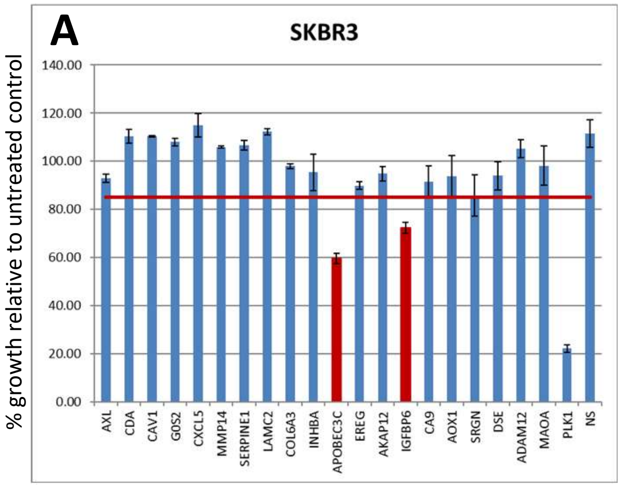


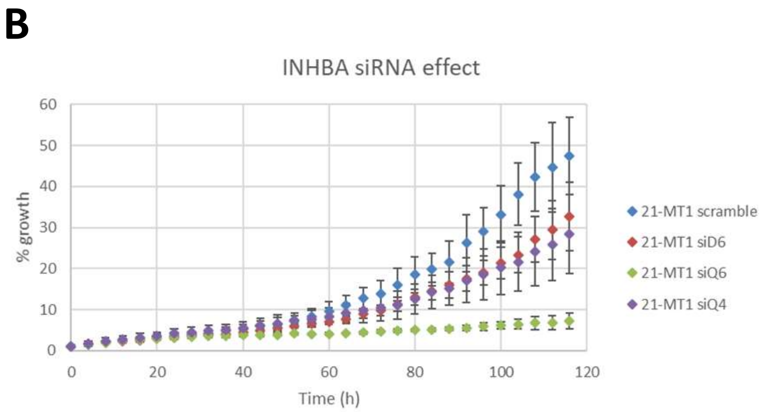


**Supplemental Figure 1A.** Knockdown of top 20 differentially expressed genes between lapatinib resistant and sensitive HER2+ cell lines in the sensitive cell line SKBR3. Knockdown of APOBEC3 and IGFBP6 inhibit growth, but knockdown of INHBA has no appreciable effect on the growth of these cells. **1B.** Use of three different siRNA's targeting INHBA in 21MT1 cells show significant impairment of growth following knockdown compared to scramble control. Error bars are +/- standard deviation.

A

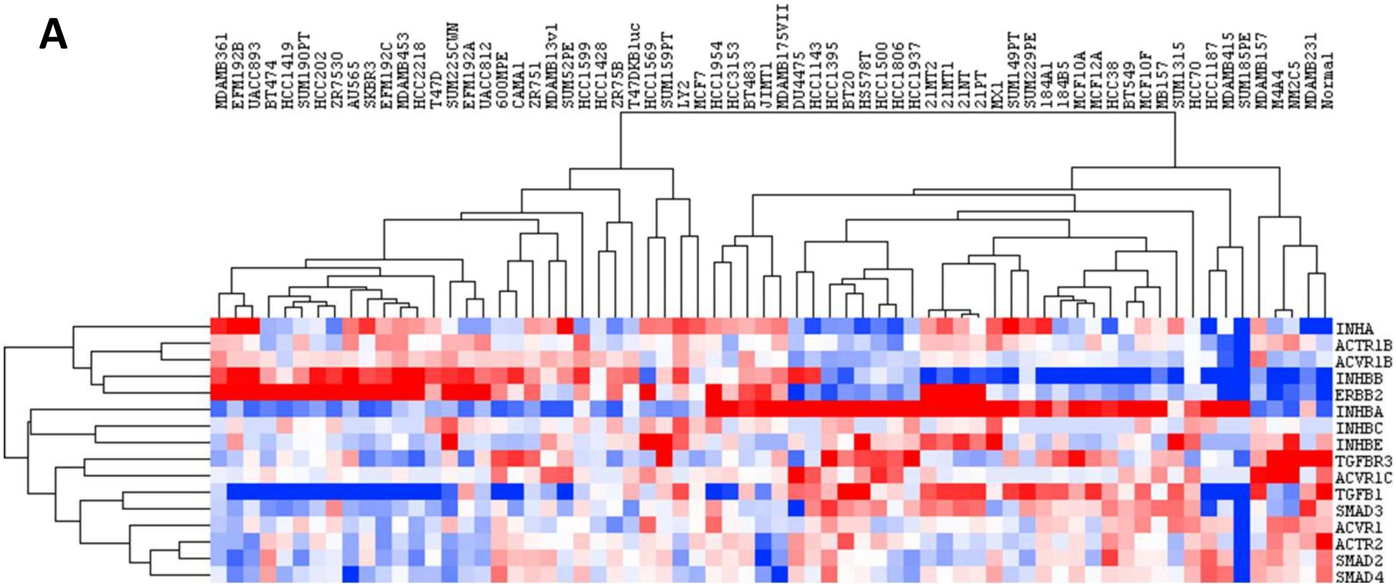


B

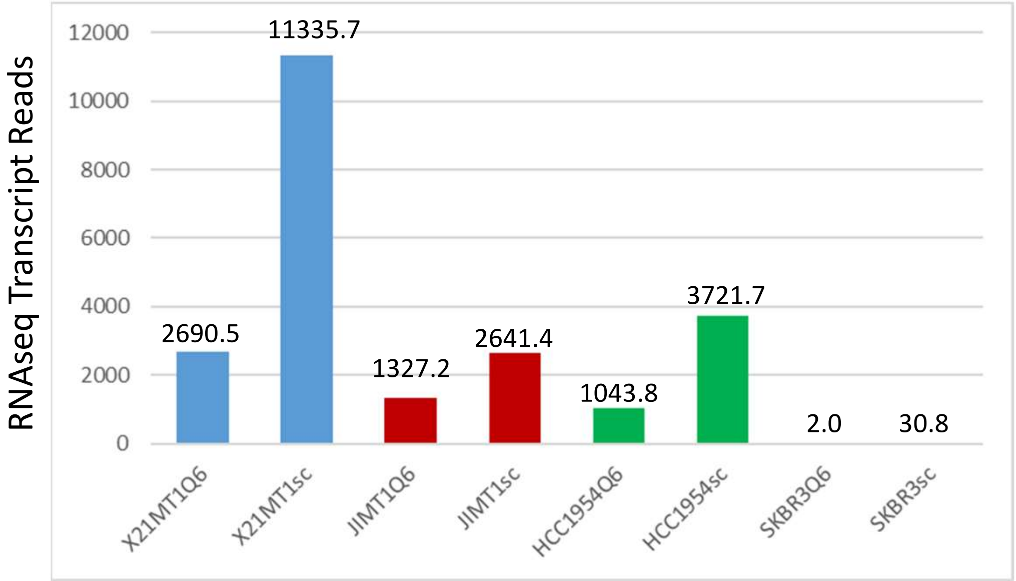


**Supplemental Figure 2. A.** Expression of INHBA and related family members in a panel of breast cancer cell lines. Note the expression of INHBA and INHBB are largely inversely correlated. **B.** RNAseq read counts as a measure of expression of INHBA in HER2+ breast cancer cells treated with INHBA siRNA (Q6) or control (sc) siRNA. INHBA siRNA treated cells show INHBA expression levels less than half of the scramble treated control cells. Note 21-MT1, JIMT1, and HCC1954 are all basal subtype cells, while SKBR3 is a luminal subtype cell. SKBR3 expression was too low to be seen on the graph. Read count values are displayed for all cells above the bars.

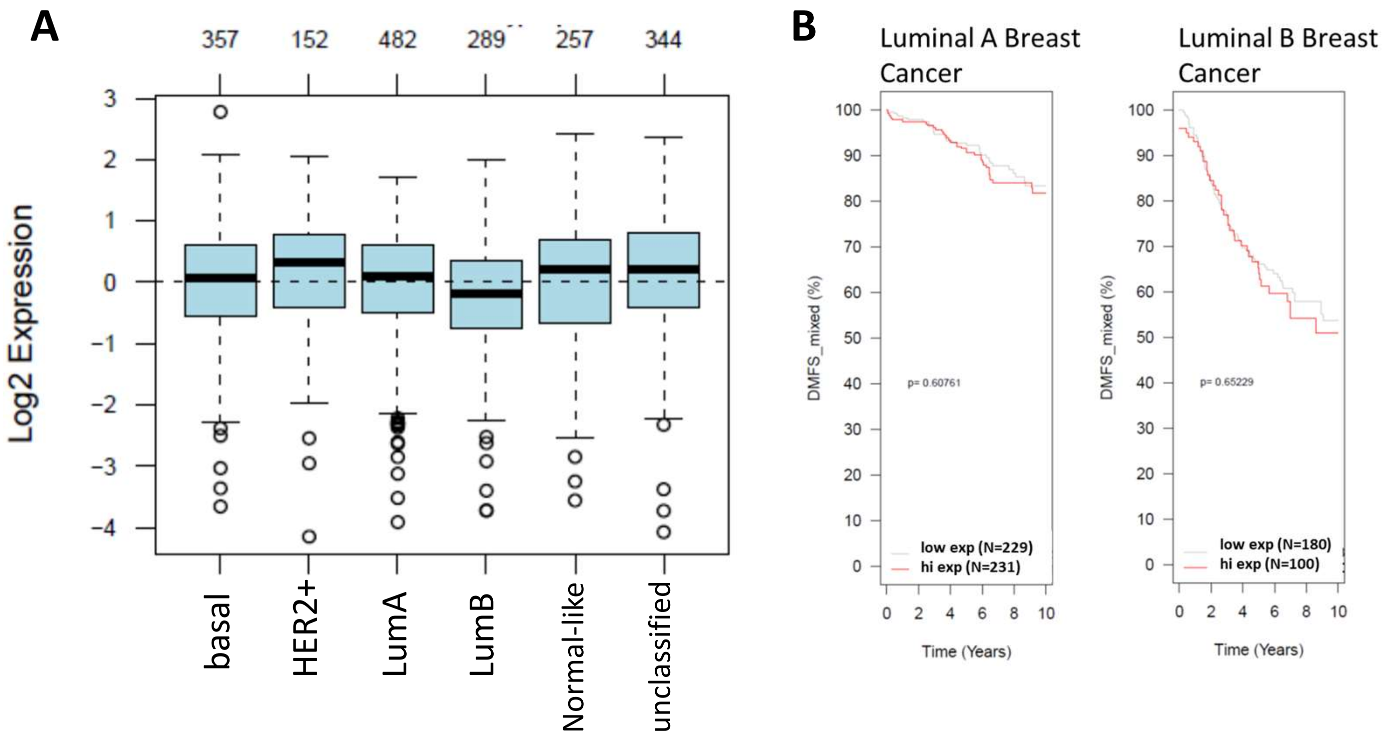


**Supplemental Figure 3A**. Boxplots showing INHBA expression in different subtypes of breast cancer from the GOBO database. Values above represent number of samples within each class. **B**. Expression of INHBA and outcome in patients with luminal A and luminal B subtype breast cancer.
